# Treatment Outcomes of Childhood Medulloblastoma with the SIOP/UKCCSG PNET-3 Protocol

**DOI:** 10.1007/s12098-023-04675-w

**Published:** 2023-06-19

**Authors:** İbrahim Kartal, Ayhan Dağdemir, Oğuz Salih Dinçer, Hülya Kangal Şimşek, Alper Uygun, Şükriye Bilge Gürsel

**Affiliations:** 1 Division of Pediatric Hematology and Oncology, Department of Pediatrics, Faculty of Medicine, Ondokuz Mayıs University, Samsun, Turkey; 2 Department of Radiation Oncology, Faculty of Medicine, Ondokuz Mayıs University, Samsun, Turkey

**Keywords:** Medulloblastoma, Treatment, Prognosis, Childhood, Radiotherapy timing

## Abstract

**Objectives:**

To retrospectively compare the overall and event-free survival rates of patients with standard and high risk medulloblastoma who received postoperative radiotherapy (RT) followed by maintenance chemotherapy.

**Methods:**

The study included 48 patients with medulloblastoma who were treated and followed-up between 2005 and 2021. Patients were classified according to the Chang classification because no molecular analysis was done. Immediately after surgery all patients received postoperative RT followed by eight cycles of chemotherapy (SIOP/UKCCSG PNET-3 protocol); if thrombocytopenia developed, carboplatin was replaced by cisplatin to avoid treatment delay. The clinical characteristics, risk categories and treatment outcomes of all patients were analyzed.

**Results:**

The mean age of the 48 patients (26 males, 22 females) at diagnosis was 7.27±4.21 y. The median start time of RT after surgery was 37 (range 19–80) d. The median follow-up was 56 (3–216) mo. The 5-year event-free survival was 61.2±10% in the high-risk group and 82.5±11.5% in the standard-risk group. The 5-year overall survival was 73.2±7.1%; it was 61.2±10% and 92.9±6.9% for high- and standard-risk patients, respectively (*p* = 0.026).

**Conclusions:**

The outcomes of patients who were started on the modified SIOP/UKCCSG PNET-3 chemotherapy protocol, in which RT was begun as soon as possible after surgery, were comparable to those of current treatment protocols. Although a definitive conclusion is difficult, given the limited number of patients in the present study, authors suggest that their treatment protocol is a viable option for centers with limited facilities (such as an inability to perform molecular analysis).

## Introduction

Medulloblastoma (MB) is the most common malignant brain tumor in children [1], and comprises about 20% of all childhood central nervous system tumors. The annual incidence is 5.07 children per million, with bimodal peaks at 3–4 and 7–8 y of age [2]. Patients younger than 3 y of age, with metastasis at diagnosis, or residual tumors >1.5 cm^2^ in the area are considered high risk [3]. Recently, large cell/anaplastic histopathology and MYC amplification have been identified as high-risk factors, while WNT over-expression has the best prognosis [4]. Here, authors present the clinical characteristics, risk categories, treatments, and outcomes of 48 MB patients who received the SIOP/UKCCSG PNET-3 chemotherapy protocol between 2005 and 2021 in their institution.

## Material and Methods

Forty-eight patients diagnosed with MB between 2005 and 2021 who were treated and followed at authors’ institution were reviewed; the last date of data collection was 12 July, 2022. The clinical features, histopathology, treatment modalities, prognostic criteria, and survival rates were analyzed. Primary tumors were evaluated by brain magnetic resonance imaging (MRI). Spinal involvement was explored using spinal MRI preoperatively or 3 wk postoperatively. Cerebrospinal fluid (CSF) was analyzed at least 3 wk postoperatively. The Chang staging system was used: M0 indicates no evidence of gross residual tumor or metastasis, M1 is the presence of microscopic tumor cells in the cerebrospinal fluid, M2 is gross nodular seeding within the central nervous system other than the spinal space, M3 is gross nodular seeding in the spinal subarachnoid space, and M4 is metastasis outside the cerebrospinal axis [5]. The extent of resection and any residual tumor were evaluated by postoperative MRI. Gross/total resection was defined as the absence of a visible tumor on postoperative imaging; subtotal resection was defined as when over 50% of the tumor was removed. The histological subtypes were those of the World Health Organization, but authors were not able to perform molecular classification [6]. Patients with postoperative residual tumors (>1.5 cm^2^ in area), who were younger than 3 y of age, and metastasis staging ≥M1 (M_risk_) were considered to be at high risk.

**Fig. 1:**
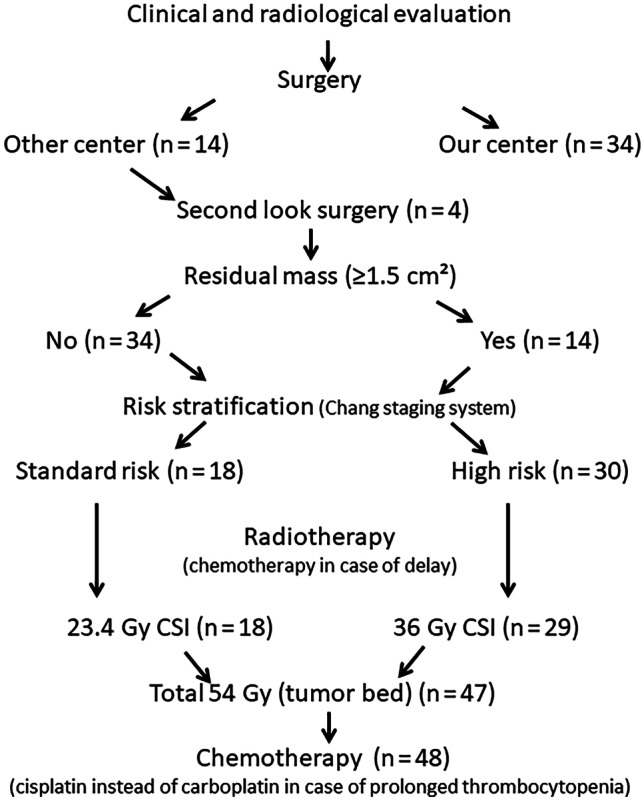
Patient management diagram

After surgery, all patients older than 3 y of age were prescribed fractionated, intensity modulated external radiotherapy (RT) to both the cranium and spinal cord commencing 3–7 wk after surgery. The craniospinal irradiation (CSI) doses were 23.4 Gy for standard and 36 Gy for high-risk patients and boost doses were delivered to a total of 54 Gy to the primary tumor bed. Radiotherapy was not given to children under 3 y of age; in these children, chemotherapy continued until 3 y of age. If a child received the total chemotherapy protocol dose, temozolomide 200/mg/m^2^/d was given for 5 d every 4 wk until radiotherapy. Vincristine was administered weekly during RT. Adjuvant chemotherapy was commenced in all patients (both standard and high-risk) approximately 2–3 wk after the end of RT; eight courses of the SIOP/UKCCSG PNET-3 protocol were delivered at an interval of at least 3 wk. In authors’ institution, based on the decision of the multidisciplinary tumor council, the timing of radiotherapy was earlier than in the original protocol; in patients with severe thrombocytopenia, 70 mg/m^2^ cisplatin was substituted for carboplatin in each course to avoid treatment prolongation (Fig. 1). In patients who relapsed or progressed, “8 drugs in a day” were usually used as salvage chemotherapy and other alternatives.

The chemotherapy protocol was conducted over four alternate cycles at a 3-wk interval (eight cycles). The regimen consisted of vincristine 1.5 mg/m^2^, given three times weekly; etoposide 100 mg/m^2^/d, given for three consecutive days; and carboplatin 500 mg/m^2^/d, given on the first two days or vincristine 1.5 mg/m^2^, given three times weekly; etoposide 100 mg/m^2^/d, given for three consecutive days; and cyclophosphamide 1.5 g/m^2^, with mesna given on the first day. Patients who received at least one course of chemotherapy were included in the study, even if they did not complete the treatment.

The study was approved by the Medical Ethics Committee (16/12/2021, no. 2021000540). Informed consent to treat the disease was obtained from the patients’ parents.

IBM SPSS ver. 23 was used for all analyses. Overall survival (OS) was defined as the time from diagnosis to death or the day of the last checkup with the healthcare team. Event-free survival (EFS) was defined as the time from diagnosis to the first recurrence or death. Kaplan–Meier survival curves were drawn and log-rank tests performed to compare OS and EFS based on the known prognostic factors and the timing of RT after surgery. Independent mortality risk factors were analyzed by Cox’s regression. Multivariable analysis was performed for factors with a *p* value <0.1 in the univariable analyses. The level of significance was taken to be *p* <0.05.

## Results

Twenty-six (54.2%) patients were male and 22 (45.8%) were female. The mean age at diagnosis was 7.27±4.21 (median 6.5, range 1–17) y; 70.8% of the patients (n = 34) underwent their initial surgery at authors’ institution. A second surgery was performed for four patients. At diagnosis, 75% of the patients (n = 36) lacked spinal involvement. Postoperative residues (≥1.5 cm^2^) were detected in 29.2% of the patients (n = 14). The histology was classical in 72.9% (n = 35) of the patients, desmoplastic nodular in 10.4% (n = 5), and large cell/anaplastic in 16.7% (n = 8). Recurrent disease developed in 18.7% (n = 9), of whom seven died and two are alive. Of the recurrences, five were metastatic and four had local disease (two high risk, two standard risk; one survived and the other three died). The median follow-up was 56 (3–216) mo. All patients under 3 y of age at diagnosis were ultimately given radiotherapy, except one who died before 3 y of age while on chemotherapy.

The 5-year event-free survival of those younger (n = 5) and older (n = 43) than age 3 was 30.0±23.9% and 74.2±7.7%, respectively (*P* = 0.038). The median start time of radiotherapy after surgery was 37 (range 19–80) d for patients >3 y old. Radiotherapy was interrupted for more than 1 wk in one patient only (now alive). The 5-year event-free survival of patients for delayed (beyond 7 wk) and non-delayed radiotherapy was 59.7±14.6% and 76.7±7.4% (*P* = 0.385), respectively, without considering the risk group. Table 1 summarizes the clinical characteristics and EFS of the patients.

**Table 1:** Clinical characteristics and event-free survival (EFS)

Characteristics	n	5-year EFS	*P* value
Age (years)			0.038
<3	5	30±23.9	
≥3	43	74.2±7.7	
Residue (cm^2^)			0.090
>1.5 cm^2^	14	57.1±16	
≤1.5 cm^2^	34	75.1±8.4	
Postoperative radiotherapy time (weeks)			0.385
>7	16	59.7±14.6	
≤7	31	76.7±8.7	
Metastasis			0.551
M0	19	76±12.3	
M1	4	75±21.7	
M2	13	69.2±12.8	
M3	12	59.5±16.2	
Overall			0.072
High risk	30	61.2±10	
Standard risk	18	82.5±11.5	

*EFS* Event-free survival

**Fig. 2:**
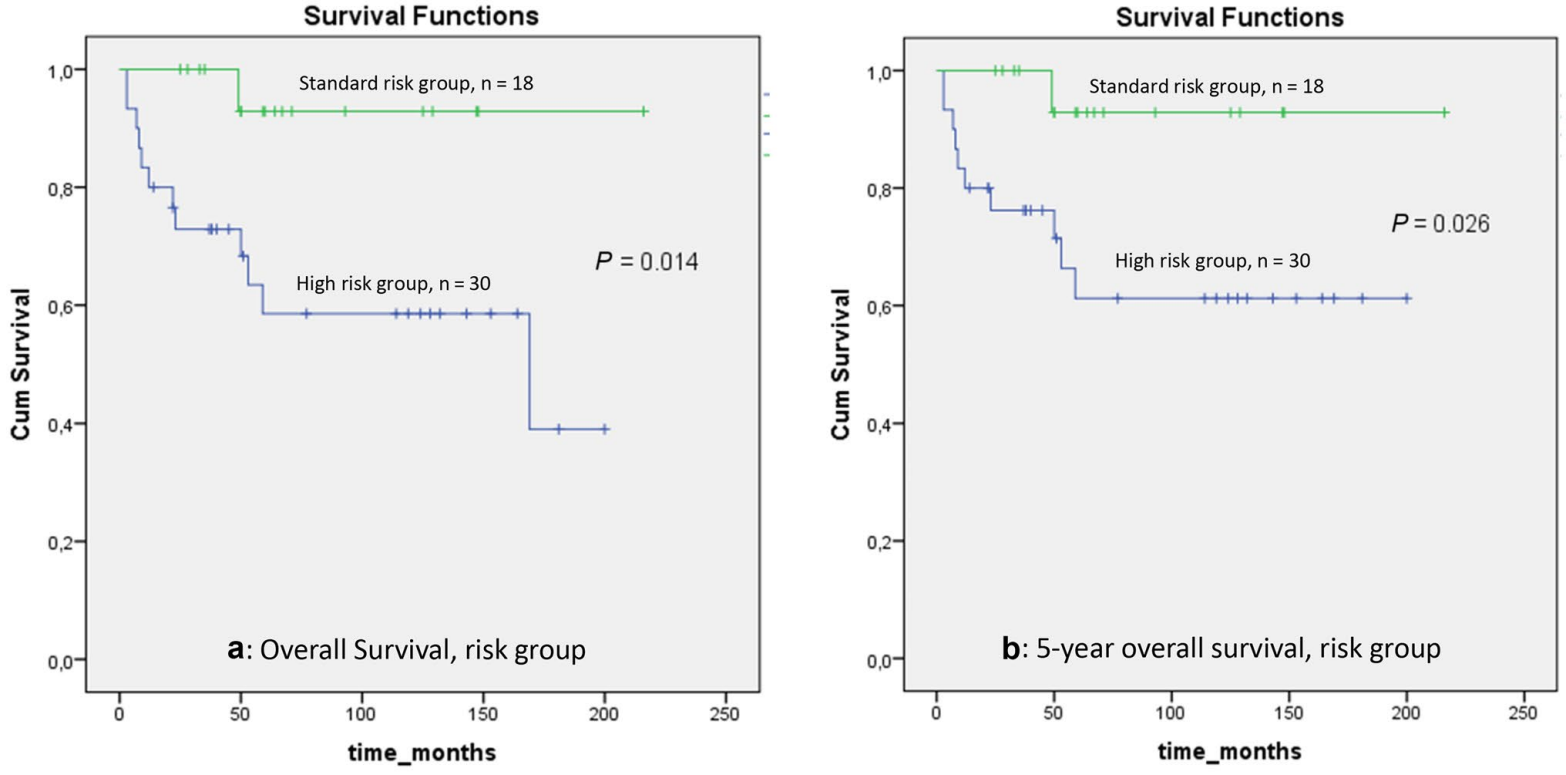
Overall (**a**) and 5-year (**b**) survival of the patients by risk group

Overall, 13 of 48 the patients died and 35 are still alive (72.9%); 12 of 30 high-risk patients and 1 of 18 standard-risk patients died. Kaplan–Meier analysis revealed that the overall survival rate was 53.6±16.4%, including 39.0±17.3% for the high-risk and 92.9±6.9% for the standard-risk patients (*P* = 0.014) (Fig. 2a). Overall 5 y, 73.2±7.1% of the patients survived: 61.2±10% for the high-risk group and 92.9±6.9% for the standard-risk group (*P* = 0.026) (Fig. 2b). The overall EFS at 5 y was 69.7± 7.6%: 61.2±10% in the high-risk group and 82.5±11.5% in the standard-risk group (*P* = 0.072).

Although authors found no significant risk factor effecting survival on univariate Cox regression analysis, the values for residual disease and age risk were borderline (*P* <0.1). On multivariable analysis, neither residual disease nor age risk were not significant but the value was *P* <0.1 for residual disease (Table 2).

**Table 2:** Independent risk factors affecting overall survival (Cox regression analysis)

Univariable (Cox)	Exp(B) (%95 CI)	*P* value
Gender		
Male	0.823 (0.273–2.477)	0.728
Female		
Spinal involvement		
Yes	2.069 (0.676–6.329)	0.203
No		
Residue		
>1.5 cm^2^	2.989 (0.986–9.064)	0.053
≤1.5 cm^2^		
Metastasis		
≥M1	0.795 (0.264–2.396)	0.684
M0		
Age (year)		
<3	3.532 (0.953–13.091)	0.059
≥3		
Timing of radiotherapy (week)		
>7	0.687 (0.217–2.170)	0.522
≤7		
Large cell/anaplastic histology		
Yes	0.038 (0–32.165)	0.342
No		

Multivariable (Cox)	Exp(B) (%95 CI)	*P* value
Residue		
>1.5 cm^2^	2.729 (0.884–8.431)	0.081
≤1.5 cm^2^		
Age (year)		
<3	3.028 (0.802–11.432)	0.102
≥3		

Three of the 35 surviving patients were followed-up in another hospital; another five were over the age of 21 and had to be followed up at adult centers. The remaining 27 patients were followed regularly. One year after treatment cessation, authors began to check for ototoxicity and detected it in five patients (two at standard risk, three at high risk); it was severe in three and mild in two. One patient had bilateral mild mixed hearing loss and this patient also had Apert syndrome. Both tympanic membranes were perforated in one patient due to chronic otitis, and he had conductive hearing loss severe enough to require a hearing aid; he underwent tympanoplasty. All the patients had neurology referrals, while there was no routine neurocognitive follow-up. The median number of episodes of febrile neutropenia was 3 (0–8) (grade 3/4) and 11 patients did not have any episodes of febrile neutropenia; no one died of febrile neutropenia. Nine patients did not require thrombocyte transfusion and the median transfusion frequency was 3 (0–7) (grade 3/4). No severe bleeding arose due to thrombocytopenia. Table 3 summarizes the adverse effects detected during follow-up.

**Table 3:** Side effects seen during patient follow-up

Side effects	n	%
Alive on follow-up	27	
Ototoxicity	5	18.5
SNHL	3	
CHL	1	
MHL + Apert syndrome	1	
Infertility (Azospermia)	3	11.1
Hypogonadotropic hypogonadism	3	11.1
Central hypothyroidism + Growth hormone deficiency	4	14.8
Panhypopituatiarism	1	3.7
Central hypothyroidism	2	7.4
Epilepsy	2	7.4
+ home MV, tracheostomy, PEG	1	
+ Urinary incontinence	1	

*CHL* Conductive hearing loss, *home MV* Home mechanical ventilator, *MHL* Mixed hearing loss, *PEG* Percutaneous endoscopic gastrostomy, *SNHL* Sensorineural hearing loss

## Discussion

The five medulloblastoma subgroups are defined histopathologically: classical, desmoplastic/nodular, extensive nodular MB, large cell, and anaplastic (the last two were subsequently combined into a single histopathological category) [7]. Medulloblastomas have also been categorized into four molecular subtypes: WNT, SHH, Group 3, and Group 4, according to the clinical and genetic features [8, 9]. As authors could not perform molecular analyses in their patients, they used the Chang Staging System for risk stratification.

It has been shown that the addition of chemotherapy to radiotherapy and completion of RT within 50 d after inception are independent predictors of improved EFS in non-metastatic patients with SIOP/UKCCSG PNET-3 in a multivariable analysis [10]. In a study of HIT-SIOP PNET-4 [11], the median time from diagnosis to RT was 37 d and the prognosis was poorer in patients in whom RT was delayed for over 7 wk. Excellent survival rates were reported in patients with no postoperative residual tumor and no delay in RT. In the present study, authors used SIOP/UKCCSG PNET-3 and initiated radiotherapy a median of 37 d after surgery, together with weekly vincristine. The survival of the present high-risk patients was similar to that reported, while the survival of standard-risk patients was > 80%, consistent with the results of the SIOP PNET-4 study [11]. There were no significant risk factor on multivariable analysis, however, if there were large number of patients, residual disease might be a candidate to be a risk factor because of borderline value (*P* = 0.081).

In metastatic MB (M2/3) patients who received SIOP/UKCCSG PNET-3 chemotherapy, the OS was 50% at 3 y and 43.9% at 5 y. The EFS was 39.7% at 3 y and 34.7% at 5 y. In this study, the median interval from surgery to RT was 117 (mean 121, range 29–212) d [12]. In the present cases, the mean start of postoperative RT was 37 d after surgery. The 5-year EFS of high-risk patients was 61.2%, while the 5-year EFS of M3 patients with only spinal involvement was 59.5%. These results are better than those of the original chemotherapy protocol, and similar to those of more recent studies in which RT was begun immediately [11, 13].

In developing countries, limited resources, the level of patient awareness, and difficulties in accessing treatment are the main causes of the failure of optimal treatment [14]. To prevent this, the patients in the present study were closely followed; the pediatric oncologists in authors’ institution follow patients on their own personal phones and are in close communication with the patients’ parents to ensure that they come to treatment regularly and undergo optimal management of their complications. When there are difficulties, families can get social assistance from the government. In Table 4, the current treatment outcomes in some centers in developing countries that treat patients without molecular analyses are summarized [15–27]. Some of these centers mention limited resources regarding treatment access [15, 17, 18, 23, 24]. Some focused on the negative consequences of patients refusing or abandoning treatment [23, 25]. Some centers point out the general problems with malnutrition, infection, and parasitic diseases in developing countries [17, 27], even when all treatment conditions are optimal.

**Table 4:** Published reports on pediatric medulloblastoma from developing countries

Author	Country	Year	n	Survival
Ali et al. [15]	Egypt	2019	53	5-year OS 54.6%, DFS 74.8%
Sirachainan et al. [16]	Thailand	2018	23	5-year OS 41.8%, DFS 60.0% (high risk)
Mehrvar et al. [17]	Iran	2018	126	7-year OS 59%, PFS 53.8%
Bleil et al. [18]	Brazil	2019	69	5-year OS 44.5%, EFS 36.4%
Muzumdar et al. [19]	India	2011	365	5-year PFS: 73% (average risk), 34% (high risk)
Gupta et al. [20]	India	2012	20	3-year relapse-free survival: 83% (average risk, age >5 y)
Kumar et al. [21]	India	2015	31	3-year OS: 40%
Gaur et al. [22]	India	2015	58	91% alive at 1.5 y
Wang et al. [23]	China	2016	67	3-year OS: 55.1%, PFS: 45.6%
Rajagopal et al. [24]	Malaysia	2017	43	5-year OS: ≥3-y-old 41.7%, <3-y-old 45.6% (high risk)
Das et al. [25]	India	2019	26	4-year EFS: 100% (average risk), 63% (high risk)
Bokun et al. [26]	Serbia	2018	87	5-year OS: 66.2%
Küpeli et al. [27]	Türkiye	2020	84	5-year OS: 58.1%, EFS: 57.6%
Kartal et al. (Current study)	Türkiye	2022	48	5-year OS: 73.2%, EFS: 69.7%

*DFS* Disease-free survival, *EFS* Event-free survival, *OS* Overall Survival, *PFS* Progression-free survival

Ototoxicity is an important risk considering the RT doses used in MB protocols; RT and cisplatin may have synergistic toxic effects. Approximately 40–60% of long-term survivors of childhood MB experience moderate to severe hearing loss [28]. In a meta-analysis of 5077 individuals, the prevalence of ototoxic hearing loss associated with carboplatin-only regimens was 13.47% (approximately one-third of that associated with cisplatin alone) [29]. Comparing intensity modulated radiotherapy (IMRT) and standard CSI, IMRT was found to give a reduced dose to the cochlea and grade 3/4 hearing loss occurred in only 13% of the IMRT group (median follow-up 18 mo) compared to 64% of the other group (median follow-up 51 mo) [28]. IMRT treatment has been used in authors’ institution for about 10 y. In present patients, the ototoxicity rate was 18.5% in the patients (5/27) followed up: one patient developed ototoxicity and additional complications after surgery; another had Apert syndrome, which has a deafness component; two had received cisplatin instead of carboplatin due to refractory thrombocytopenia; and one had no risk factors. The lower ototoxicity in present patients might be due to the use of carboplatin and IMRT, but the frequency could increase over time with follow-up.

This study has some limitations. It was a single center study with a small number of patients. The authors could not perform molecular analysis and had to group the patients according to the Chang system. There were no neurocognitive evaluations and it was not a randomized prospective study. The authors have begun to refer their patients for molecular analysis to a feasible center and are considering as part of the risk stratification for the same treatment protocol. As patient survival increases, the long-term effects will become more important, like short stature, infertility, and neurocognitive dysfunction.

In summary, the authors prioritized the RT timing of the original SIOP/UKCCSG PNET-3 protocol and changed carboplatin to cisplatin in patients with thrombocytopenia to eliminate any treatment delay in patients with medulloblastoma. The patients’ outcomes were similar to those of current protocols using molecular studies. Although a definitive conclusion is impossible given the small number of patients, authors suggest that the present protocol is a viable option for centers with limited facilities.
